# To MRAs treatment or not? evidence from a meta-analysis of randomized controlled trials of different MRAs on cardiovascular health in heart failure

**DOI:** 10.3389/fcvm.2025.1564860

**Published:** 2025-07-23

**Authors:** Jinyu He, Gang Chen, Yujia Huo, Chunyu Zhang, Haojie Xue, Jing Wang, Yi Zhong, Juan Xiao, Hongping Shen, Jian Feng

**Affiliations:** ^1^ Department of Cardiology, The Affiliated Hospital of Southwest Medical University, Luzhou, China; ^2^ Clinical Pharmacy Office, The Affiliated Traditional Chinese Medicine Hospital of Southwest Medical University, Luzhou, China; ^3^ Department of Cardiology, Southwest Medical University Affiliated Hospital Medical Group Gulin Hospital (Gulin County People’s Hospital), Southwest Medical University, Luzhou, China

**Keywords:** heart failure, mineralocorticoid receptor antagonists, all-cause mortality, cardiovascular death, meta-analysis

## Abstract

**Background:**

Mineralocorticoid receptor antagonists (MRAs) are pivotal in heart failure (HF) management.

**Objectives:**

This study evaluates their impact on adverse cardiovascular events and left ventricular ejection fraction (LVEF) in HF patients.

**Methods:**

A comprehensive literature search was conducted across PubMed, Embase, Cochrane Library, Web of Science, and ClinicalTrials databases, with a cutoff date of September 30, 2024. All included studies were randomized controlled trials (RCTs) that recorded the incidence of adverse cardiovascular events and changes in LVEF after MRA treatment in HF patients.

**Results:**

A total of 30 randomized controlled trials involving 24,831 patients with heart failure were included. Compared to conventional therapy or placebo, treatment with MRAs significantly reduced the risk of all-cause mortality (RR = 0.862, 95% CI: 0.778–0.956, *p* = 0.005; *I*^2^ = 36.1%), cardiovascular mortality (RR = 0.828, 95% CI: 0.732–0.937, *p* = 0.003; *I*^2^ = 45.7%), and heart failure-related hospitalization (RR = 0.780, 95% CI: 0.657–0.926, *p* = 0.005; *I*^2^ = 65.5%). Moreover, MRAs significantly improved LVEF (WMD = 1.384, 95% CI: 0.208–2.559, *p* = 0.021; *I*^2^ = 59.9%). However, MRA therapy was associated with an increased risk of renal dysfunction, including hyperkalemia (RR = 2.086, 95% CI: 1.872–2.325, *p* < 0.001; *I*^2^ = 0.0%), elevated serum creatinine (RR = 1.512, 95% CI: 1.252–1.825, *p* < 0.001; *I*^2^ = 0.0%), decreased eGFR (WMD = −5.223, 95% CI: −7.380 to −3.066, *p* < 0.001; *I*^2^ = 0.0%), and potentially increased incidence of composite renal outcomes.

**Conclusion:**

MRAs significantly reduce the risk of adverse cardiovascular events in patients with heart failure and contribute to LVEF improvement. They lower all-cause mortality in patients with HFrEF and reduce hospitalization for heart failure in those with HFmrEF or HFpEF. However, the potential risk of renal-related adverse events warrants close monitoring.

Our protocol was registered in PROSPERO (registration number: CRD42024592012).

## 1 Introduction

Heart failure (HF) is a complex clinical syndrome caused by structural and/or functional abnormalities of the heart, typically accompanied by elevated natriuretic peptides and systemic fluid retention, severely impacting patients' quality of life (1). Heart failure has become a global epidemic; according to the 2017 global heart failure survey, approximately 64.3 million individuals worldwide are affected (2). With the ongoing aging population, this number is expected to rise, further exacerbating the societal health and economic burden (3).

Mineralocorticoid receptors (MR) are nuclear receptors, and their overactivation can lead to a series of pathological processes, including inflammation, fibrosis, and oxidative stress, which contribute to the progression of heart failure. Aldosterone, the physiological ligand for MR, is significantly elevated in heart failure patients, leading to pathological remodeling of the myocardium and vasculature. Mineralocorticoid receptor antagonists (MRAs) mitigate these pathological processes by blocking MR, playing a critical role in heart failure management (4).

In 1999, Pitt et al. demonstrated in a clinical trial (5) that spironolactone significantly reduces mortality risk in patients with heart failure with reduced ejection fraction (HFrEF) compared to placebo. In 2011, a large randomized controlled trial showed that eplerenone significantly decreases the risk of mortality and hospitalization in heart failure (HF) patients (6). The TOPCAT study suggested that spironolactone (an MRA) may offer potential benefits for patients with heart failure with preserved ejection fraction (HFpEF) (7). However, the efficacy and safety of MRAs in heart failure remain controversial. Early animal studies by Young et al. (8) revealed that mineralocorticoid receptor agonists could induce inflammatory responses and organ fibrosis, further exacerbating target organ damage. Additionally, Yancy CW et al. highlighted the uncertain efficacy of MRAs in patients with heart failure with mildly reduced ejection fraction (HFmrEF) and HFpEF. Currently, HFmrEF/HFpEF patients lack standardized treatment options, and managing this population remains highly contentious (9).

Current guidelines recommend the use of mineralocorticoid receptor antagonists (MRAs), particularly spironolactone and eplerenone, to reduce adverse cardiovascular events in heart failure (5, 6, 10).Emerging evidence has shown that a novel nonsteroidal selective MRA, finerenone, exhibits more pronounced anti-inflammatory and antifibrotic effects (11). Two large studies demonstrated that finerenone effectively reduces the risk of cardiovascular events in patients with chronic kidney disease or diabetes (12, 13). Its efficacy and safety in HFmrEF/HFpEF are currently under extensive investigation. The recently published global Phase III trial (FINEARTS-HF) (14) on finerenone provides robust evidence for the use of MRAs in heart failure. Previous systematic reviews of MRAs in randomized trials yielded inconsistent findings regarding their role in reducing adverse cardiovascular outcomes (15, 16), highlighting the need for additional evidence to clarify their benefits in heart failure patients.

This meta-analysis included 30 randomized controlled trials, incorporating nearly all available primary data on the use of mineralocorticoid receptor antagonists (MRAs) in heart failure. The primary objective was to assess the impact of MRAs on adverse cardiovascular outcomes—specifically all-cause mortality, cardiovascular mortality, heart failure hospitalization, and improvement in left ventricular ejection fraction (LVEF). Additionally, the study investigated the risk of renal adverse events potentially associated with MRA therapy, including hyperkalemia, increased serum creatinine, decreased estimated glomerular filtration rate (eGFR), the composite renal outcome. The analysis also explored differences in treatment effects across patient groups with varying baseline LVEF and examined whether different MRA agents (e.g., spironolactone, eplerenone, finerenone, canrenone) yielded heterogeneous cardiovascular outcomes.

## 2 Methods

This systematic review was conducted and reported in accordance with the PRISMA 2020 statement (17).

### 2.1 Search strategy

We systematically searched PubMed, Embase, Cochrane Central Register of Controlled Trials (CENTRAL), Web of Science, and Clinicaltrials.gov databases for relevant studies up to September 30, 2024. No language or publication date restrictions were applied during the search. The search strategies for each database are provided in Supplementary Table S1. After removing duplicates, two authors independently screened the titles and abstracts of the retrieved studies for initial selection. Full-text articles were further assessed for studies potentially meeting the inclusion criteria. In addition, we examined the reference lists of included studies to identify any relevant studies that might have been overlooked. Any disagreements during the screening process were resolved through discussion with a third author. For studies lacking primary data, we attempted to contact the authors to obtain the necessary information.

### 2.2 Inclusion criteria

We included randomized controlled trials (RCTs) that met the following criteria: (1) Study Population: Heart failure (HF) patients;(2) Study Design: The intervention group received MRAs (mineralocorticoid receptor antagonists) treatment, while the control group received either standard care or placebo;(3) Outcomes: The study assessed at least one of the following outcomes: all-cause mortality, cardiovascular mortality, heart failure hospitalization, hyperkalemia, changes in left ventricular ejection fraction (LVEF), the composite renal outcomes (including one or more of the following events: renal injury, deterioration of renal function (with or without hospitalization), renal-related death, etc. And the definitions follow those reported in the original studies (Supplementary Table S2).

### 2.3 Exclusion criteria

(1) Non-randomized controlled trials (e.g., cross-sectional studies, cohort studies, preclinical studies, or animal experiments); (2) Studies lacking predefined outcomes; (3) Reviews, meta-analysis, editorials, and conference abstracts; (4) Studies unrelated to the objectives of this meta-analysis.

### 2.4 Data extraction and quality assessment

Two authors independently screened the eligible studies based on predefined inclusion and exclusion criteria and conducted a comprehensive analysis of the selected studies. Data extraction was carried out independently using standardized forms, with any disagreements resolved through consultation with a thirdauthor. The extracted data included: (1) Basic information: first author, publication year, country of study, and trial registration number. (2) Study design: trial type and methodological characteristics. (3) Participant characteristics: sample size, baseline characteristics of participants, interventions, controls, and their respective doses. (4) Outcome measures: number of patients for each observed outcome. (5) Follow-up duration. (6) Results of interest. For studies with incomplete data, we supplemented the information by consulting trial registries (e.g., ClinicalTrials.gov), supplemental materials, original manuscripts, tables, and figures. The eligibility of studies for inclusion in the meta-analysis was rigorously determined based on predefined inclusion and exclusion criteria. Each study was systematically screened to ensure it met the specified criteria for participant population, intervention, and outcome measures. Key intervention characteristics were systematically tabulated and compared against the predefined groups planned for each synthesis, ensuring consistency and relevance with the study objectives. We systematically reviewed full texts and registration records to ensure only independent RCTs were included, and used the following methods to minimize duplicate participant inclusion and associated bias: ① Prioritized data from original trial publications, ensuring independence and consistency; ② For multiple versions of the same trial (e.g., reanalyses or derivative reports), only the version with the most comprehensive population and outcomes was included, while others were excluded; ③ For multi-arm trials sharing a common control, we adhered to Cochrane Handbook (v6.5, sections 23.3.2 & 23.3.4) (18) by combining intervention arms or selecting a representative arm, thus avoiding double counting and reducing undue narrowing of confidence intervals and bias.

### 2.5 Risk of bias assessment for individual studies

The risk of bias for each study was assessed using the Cochrane Risk of Bias tool (ROB2), evaluating five key domains: randomization process, deviations from intended interventions, missing outcome data, outcome measurement, and selective reporting. Each study's risk of bias was categorized as “low risk,” “some concerns,” or “high risk.”

### 2.6 Statistical analysis

Statistical analyses were performed using STATA software. Study characteristics were summarized using a structured data extraction form. Dichotomous outcomes were presented as relative risk (RR) with 95% confidence intervals (CI), while continuous outcomes were expressed as weighted mean difference (WMD) with 95% CI. Between-study heterogeneity was assessed using Cochran's *Q* test and the *I*^2^ statistic.

Forest plots were used to visually display the individual effect sizes and their 95% CIs, with the pooled estimates shown as diamonds. The degree of heterogeneity (*I*^2^) was presented at the bottom of each plot. Funnel plots were generated for visual inspection of publication bias, and Egger's test was used to provide statistical confirmation.

Preliminary meta-analyses revealed potential differences among studies in terms of patient characteristics, inclusion/exclusion criteria, and statistical approaches. Based on the latest edition of the Cochrane Handbook for Systematic Reviews of Interventions (19), we applied either fixed-effect or random-effects models according to the heterogeneity level: fixed-effect models were used when *I*^2^ < 20%, and random-effects models when *I*^2^ ≥ 20% or when mild heterogeneity was present. In cases of substantial heterogeneity, findings were interpreted with caution. For subgroup analyses with *I*^2^ near 20%, model choice was made prudently based on between-study variation, and such instances were clearly annotated. A two-tailed *P*-value <0.05 was considered statistically significant.

The certainty of evidence for each outcome was evaluated using the GRADE approach. This framework considers factors such as risk of bias, inconsistency, imprecision, indirectness, and publication bias to rate the quality of evidence as high, moderate, low, or very low. Risk of bias was assessed using the ROB2 tool, while heterogeneity was quantified with *I*^2^ statistics. Publication bias was examined through funnel plots and Egger's test. Any concerns related to these domains resulted in downgrading the quality of evidence for specific outcomes.

### 2.7 Sensitivity and subgroup analyses

To assess the robustness of the results, we conducted a sensitivity analysis using a “leave-one-out” approach to examine the impact of each individual study on the overall results. Additionally, the following subgroup analyses were performed: (1) Based on baseline LVEF, studies were grouped into LVEF < 40% (HFrEF) and LVEF ≥ 40% (HFmrEF/HFpEF) to explore differential treatment effects. Studies lacking LVEF data or presenting conflicting classifications were excluded from this analysis.; (2) Based on age: The patients were categorized into two groups, those aged <65 years and those aged ≥65 years, to examine the efficacy of MRAs in different age groups. Studies without reported age data were classified into the “unknown age group”; (3) Based on drug type: Analyses were conducted based on different types of MRAs (e.g., spironolactone, eplerenone, finerenone, canrenone) to investigate the impact of different drugs on cardiovascular outcomes.

## 3 Results

**Figure 1:**
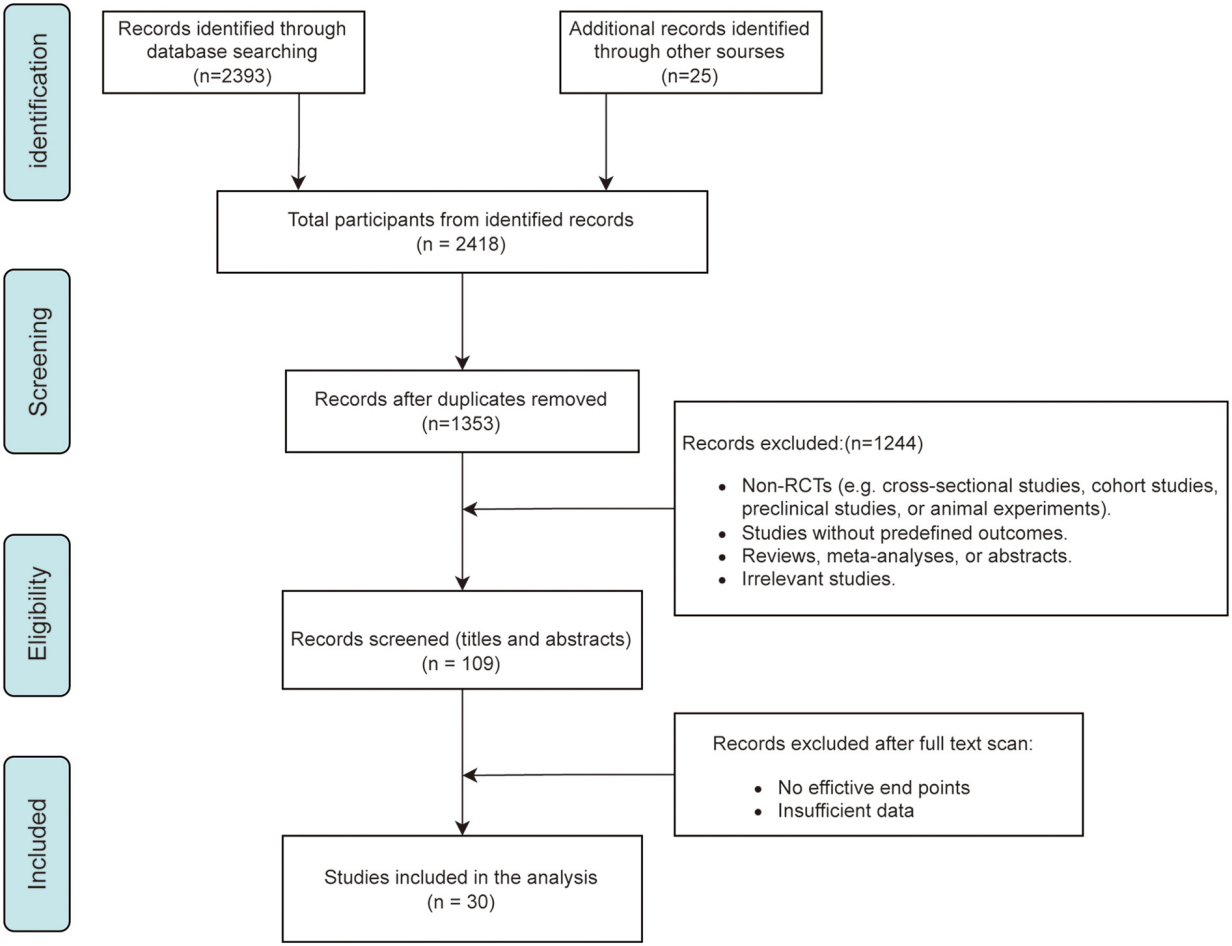
Flowchart of literature search and study selection.

The literature screening process is shown in Figure 1. A total of 2,418 potential records were identified through systematic searches, and after removing duplicates, 1,353 articles remained. Following an initial screening of titles and abstracts, 109 studies were included for full-text review. Based on predefined outcome events, 30 randomized controlled trials (5–7, 10, 14, 20–44) were ultimately included, involving a total of 24,831 participants. Detailed study characteristics are presented in Table 1. Among the included studies, 20 trials (5, 7, 20, 21, 24–26, 28–30, 32–35, 39–44) used spironolactone, 8 trials (6, 10, 22, 27, 31, 36–38) used eplerenone, 2 trials (14, 35) used finerenone, and 1 trial (23) used canrenone. The studies were grouped according to baseline population characteristics, including mean age, baseline LVEF, and type of MRAs used, to assess efficacy differences across subgroups (Table 2).

**Table 1:** Characteristics for included patients and relevant outcomes.

Author	Year	Nation	Characteristics	Participants (n)	Age (years) (mean ± SD)	Male (%)	Type of intervention	Dose	Endpoints	Follow-up duration
Akbulut et al.	2003	Turkey	NYHA III; LVEF ≤ 35%; K < 5.5 mmol/L	70	59.1 ± 5.7	55.7	Spironolactone	25 mg/day	LVEF, Hyperkalemia	3 months
Asakura et al.	2022	Japan	AHF; LVEF ≤ 40%; ≥20 years	300	67.5 ± 3.5	72.7	Eplerenone	25–50 mg/day	ACM, CVD, HHF, LVEF, Hyperkalemia, Composite Renal Outcome	6 months
Boccanelli et al.	2009	Italy	NYHA II; LVEF ≤ 45%; 18–80 years	467	62.5 ± 9.5	83.5	Canrenone	25–50 mg/day	ACM, CVD, HHF, LVEF, Hyperkalemia	12 months
Chan et al.	2007	China	LVEF < 40%; ACEI for more than 6 months	48	63.3 ± 8.6	83.3	Candesartan & Spironolactone	8 mg/day candesartan & 25 mg/day spironolactone	Hyperkalemia	13 months
Chen et al.	2016	China	DHF; LVEF ≥ 50%	93	75.8 ± 6.6	NA	Furosemide & Spironolactone	20 mg/day furosemide & 40 mg/day spirolactone or 40 mg/day furosemide & 100 mg/day spirolactone	HHF, LVEF	1 months
Cicoira et al.	2002	Italy	CHF; LVEF ≤ 45%; patients with sinus rhythm only	106	NA	86.8	Spironolactone	25–50 mg/day, 12.5 mg/day if hyperkalemia	LVEF, Hyperkalemia	12 months
Deswal et al.	2011	USA	DHF; NYHA II; LVEF ≥ 50%; ≥18 years; BP ≤ 150/95mmHg; BNP ≥ 100pg/ml	44	70.4 ± 9.5	93.2	Eplerenone	25 mg/day for 2 weeks, if tolerance 50 mg/day for 22 weeks	HHF, LVEF, Hyperkalemia, Creatinine Elevation Events	6 months
Edelmann et al.	2013	Germany	DHF; NYHA II/III; LVEF ≥ 50%	422	67 ± 8	47.6	Spironolactone	25 mg/day	LVEF, Hyperkalemia, GFR, Composite Renal Outcome	12 months
Gandhi et al.	2012	USA	HErEF; NYHA II to IV; Galectin-3 ≤ 20 ng/ml	76	57.4 ± 13.7	89.5	Spironolactone	25 mg/day (median)	CVD, HHF, LVEF, eGFR	10 months
Gandhi et al.	2012	USA	HErEF; NYHA II to IV; Galectin-3 >20 ng/ml	75	69.4 ± 11.4	78.7	Spironolactone	25 mg/day (median)	CVD, HHF, LVEF, eGFR	10 months
Gao et al.	2007	China	NYHA II–IV; LVEF < 45%	116	54.5 ± 12.5	64.7	Spironolactone	20 mg/day	LVEF, Hyperkalemia, Creatinine Elevation Events	6 months
Jha et al.	2022	India	HF; >55 years	500	NA	NA	Eplerenone	50 mg/day	ACM, CVD, HHF	6 months
Kosmala et al.	2016	Poland	NYHA II/III; LVEF > 50%; diastolic dysfunction; exertional E/e' > 13	131	67 ± 9	16	Spironolactone	25 mg/day	LVEF	6 months
Mak et al.	2009	Ireland	HFpEF; LVEF > 45%; previously admitted to NYHA IV; BNP > 100pg/ml; diastolic dysfunction	44	80 ± 7.8	45.5	Eplerenone	25 mg/day for 6 months, increase to 50 mg/day until 12 months	LVEF, BNP, Creatinine	12 months
McDiarmid et al.	2020	UK	HFpEF; NYHA II to IV; LVEF > 50%; 18–90 years; NT-proBNP > 400 pg/L	40	75.1 ± 7.3	50	Spironolactone	25 mg/day	LVEF, Creatinine	6 months
Pitt et al.	1996	USA	NYHA II–IV; LVEF ≤ 35%; congestive heart failure	214	61.8 ± 11.8	80	Spironolactone	12.5–75 mg/day	Hyperkalemia	3 months
Pitt et al.	2014	USA	LVEF ≥ 45%; symptomatic HF;≥50 years; K < 5.0 mmol/L	3445	68.7 ± 2.2	48.5	Spironolactone	15–45 mg/day	ACM, CVD, HHF, Hyperkalemia, Creatinine Elevation Events	40 months
Pitt et al.	1999	USA	NYHA III/IV; LVEF ≤ 35%	1663	65 ± 12	73.2	Spironolactone	25 mg/day, 50 mg/day if no hyperkalemia, 25 mg qod if hyperkalemia	CVD, HHF, Hyperkalemia	24 months
Pitt et al.	2003	USA	AMI; LVEF ≤ 40%	6,632	64 ± 11.5	71.1	Eplerenone	25 mg/day, increasing to 50 mg/day after 4 w	ACM, CVD, HHF, Hyperkalemia	16 months
Pitt et al.	2013	USA	HFrEF; NYHA II-III; LVEF ≤ 40%; moderate CKD	320	NA	NA	Finerenone	2.5 or 5 or 10 mg/day or 5 mg bid	HHF, BNP, Hyperkalemia, eGFR, Composite Renal Outcome	1 months
Pitt et al.	2013	USA	HFrEF; NYHA II–III; LVEF ≤ 40%; moderate CKD	76	NA	NA	Spironolactone	25 or 50 mg/day	HHF, BNP, Hyperkalemia, eGFR	1 months
Pitt et al.	2013	USA	HFrEF; NYHA II–III; LVEF ≤ 40%; mild CKD	65	NA	NA	Finerenone	2.5 mg/day or 5 mg/day or 10 mg/day	Hyperkalemia	1 months
Solomon et al.	2024	USA	LVEF ≥ 40%; >40 years; Symptoms of heart failure	6,001	71.9 ± 9.6	54.5	Finerenone	20 mg/day or 40 mg/day	ACM, CVD, Hyperkalemia, Creatinine Elevation Events, Composite Renal Outcome	32 months
Taheri et al.	2012	Iran	NYHA III–IV; LVEF ≤ 45%; K ≤ 5.5 mmol/L; CAPD	18	54 ± 15.3	NA	Spironolactone	25 mg/day	LVEF	54 months
Taheri et al.	2009	Iran	NYHA III–IV; LVEF < 45%; K < 5.5; hemodialysis	16	58.2 ± 7.9	86.8	Spironolactone	25 mg/day	LVEF, Hyperkalemia	6 months
Tsutamoto et al.	2001	Japan	CHF; NYHA II/III; LVEF < 45%	37	63.8 ± 3.3	75.7	Spironolactone	25 mg/day	LVEF, BNP	4 months
Tsutsui et al.	2017	Japan	HFrEF; NYHA II–IV; LVEF ≤ 35%	221	68.7 ± 8.2	79.6	Eplerenone	25 mg/day, K < 5 mmol/L, increased to 50 mg/day after 4 w	ACM, CVD, HHF, Hyperkalemia, Composite Renal Outcome	12 months
Udelson et al.	2010	USA	NYHA II/III; LVEF ≤ 35%; ≥21 years	226	62.7 ± 12.5	83.6	Eplerenone	25 mg/day, increase to 50 mg/day after 4 w	LVEF, BNP, Hyperkalemia, Creatinine Elevation Events	9 months
Upadhya et al.	2017	USA	HFpEF; controlled BP	80	71 ± 1	20	Spironolactone	25 mg/day	LVEF, BNP	9 months
Vizzardi et al.	2014	Italy	CHF; NYHA I or II; LVEF < 40%	130	62.2 ± 17.9	NA	Spironolactone	25–100 mg/day	ACM, CVD, Hyperkalemia, Composite Renal Outcome	7–15 months
Wu et al.	2016	China	NYHA II; LVEF ≤ 45%; 18–80 years	139	66.1 ± 1.4	50.4	Spironolactone	10–20 mg/day	ACM, CVD, HHF, LVEF, Hyperkalemia	60 months
Xin et al.	2019	China	HFmrEF(LVEF 40%–49%); NYHA ≥ II	279	64.7 ± 11.7	43.3	Spironolactone	25–50 mg/day	ACM, HHF	12 months
Zannad et al.	2011	France	NYHA II; LVEF ≤ 35%	2,737	68.6 ± 7.6	77.7	Eplerenone	25 mg/day, increase to 50 mg/day after 4 w	ACM, CVD, HHF, Hyperkalemia, Creatinine, Composite Renal Outcome	21 months

Abbreviations: ACEI, angiotensin-converting enzyme inhibitor; ACM, all-cause mortality; AHF, acute heart failure; AMI, acute myocardial infarcion; BNP, B-type natriuretic peptide; BP, blood pressure; CHF, chronic heart failure; CKD, chronic kidney disease; CVD, cardiovascular death; DHF, diastolic heart failure; HF, heart failure; eGFR, estimated glomerular filtration rate; HFrEF, heart failure with reduced ejection fraction; HFpEF,heart failure with preserved ejection fraction; HHF, hospitalization for heart failure; LVEF, left ventricular ejection fraction; NT-pro BNP, N-terminal pro-brain natriuretic peptide; RCT, Randomized Controlled Trials.

**Table 2:** Subgroup analysis of MRA effects on cardiovascular outcomes in heart failure patients.

Subgroup	Number of studies	RR/WMD	95% CI	*Z*-Test *P* Value	Heterogeneity between studies	Model Selection
All-cause mortality						
Spironolactone	4	0.682	(0.424, 1.098)	0.115	*I*^2^ = 58.4%	Random effects model
Eplerenone	5	0.851	(0.743, 0.974)	0.020	*I*^2^ = 26.3%	Random effects model
Finerenone	1	0.939	(0.839, 1.051)	0.273	—	N/A
Canrenone	1	0.519	(0.198, 1.357)	0.181	—	N/A
Age < 65 years	4	0.713	(0.472, 1.076)	0.107	*I*^2^ = 40.2%	Random effects model
Age ≥ 65 years	6	0.897	(0.780, 1.031)	0.127	*I*^2^ = 43.3%	Random effects model
Unknown age	1	0.765	(0.558, 1.049)	0.096	-	N/A
LVEF < 40%	5	0.861	(0.784, 0.947)	0.002	*I*^2^ = 21.0%	Fixed effects model
LVEF ≥ 40%	3	0.896	(0.750, 1.072)	0.230	*I*^2^ = 57.2%	Random effects model
Cardiovascular death						
Spironolactone	6	0.754	(0.560, 1.014)	0.062	*I*^2^ = 58.2%	Random effects model
Eplerenone	5	0.847	(0.703, 1.019)	0.078	*I*^2^ = 40.2%	Random effects model
Finerenone	1	0.929	(0.786, 1.099)	0.391	—	N/A
Canrenone	1	0.576	(0.196, 1.692)	0.316	—	N/A
Age < 65 years	4	0.820	(0.392, 1.714)	0.597	*I*^2^ = 55.4%	Random effects model
Age ≥ 65 years	8	0.841	(0.716, 0.987)	0.034	*I*^2^ = 53.4%	Random effects model
Unknown age	1	0.727	(0.478, 1.107)	0.138	—	N/A
LVEF < 40%	8	0.829	(0.684, 1.005)	0.057	*I*^2^ = 55.4%	Random effects model
LVEF ≥ 40%	2	0.921	(0.810, 1.049)	0.214	*I*^2^ = 0.0%	Fixed effects model
Heart failure hospitalization						
Spironolactone	8	0.880	(0.688, 1.125)	0.309	*I*^2^ = 60.7%	Random effects model
Eplerenone	6	0.738	(0.611, 0.893)	0.002	*I*^2^ = 44.3%	Random effects model
Finerenone	1	0.591	(0.123, 2.847)	0.512	—	N/A
Canrenone	1	0.368	(0.148, 0.915)	0.031	—	N/A
Age < 65 years	4	0.802	(0.551, 1.166)	0.247	*I*^2^ = 48.6%	Random effects model
Age ≥ 65 years	9	0.795	(0.623, 1.014)	0.065	*I*^2^ = 76.1%	Random effects model
Unknown age	3	0.530	(0.321, 0.875)	0.013	*I*^2^ = 0.0%	Fixed effects model
LVEF < 40%	10	0.874	(0.698, 1.095)	0.242	*I*^2^ = 71.2%	Random effects model
LVEF ≥ 40%	4	0.823	(0.702, 0.966)	0.017	*I*^2^ = 0.0%	Fixed effects model
Hyperkalemia						
Spironolactone	12	2.021	(1.709, 2.391)	0.000	*I*^2^ = 0.0%	Fixed effects model
Eplerenone	6	1.900	(1.547, 2.334)	0.000	*I*^2^ = 0.0%	Fixed effects model
Finerenone	3	2.322	(1.896, 2.844)	0.000	*I*^2^ = 0.0%	Fixed effects model
Canrenone	1	2.937	(1.341, 6.432)	0.007	—	N/A
Age < 65 years	9	1.876	(1.458, 2.414)	0.000	*I*^2^ = 0.9%	Fixed effects model
Age ≥ 65 years	9	2.132	(1.889, 2.405)	0.000	*I*^2^ = 0.0%	Fixed effects model
Unknown age	4	2.871	(0.813, 10.145)	0.101	*I*^2^ = 0.0%	Fixed effects model
LVEF < 40%	12	1.905	(1.565, 2.320)	0.000	*I*^2^ = 0.0%	Fixed effects model
LVEF ≥ 40%	4	2.167	(1.895, 2.477)	0.000	*I*^2^ = 0.0%	Fixed effects model
LVEF						
Spironolactone	15	1.353	(−0.086, 2.791)	0.065	*I*^2^ = 62.6%	Random effects model
Eplerenone	3	0.975	(−1.948, 3.899)	0.513	*I*^2^ = 43.8%	Random effects model
Finerenone	N/A	N/A	N/A	N/A	N/A	N/A
Canrenone	1	2.200	(0.262, 4.138)	0.026	—	N/A
Age < 65 years	8	1.172	(−0.019, 2.364)	0.054	*I*^2^ = 0.0%	Fixed effects model
Age ≥ 65 years	10	1.314	(−0.456, 3.085)	0.146	*I*^2^ = 73.9%	Random effects model
Unknown age	1	2.000	(−1.659, 5.659)	0.284	—	N/A
LVEF < 40%	5	0.748	(−0.747, 2.243)	0.327	*I*^2^ = 0.0%	Fixed effects model
LVEF ≥ 40%	7	0.531	(−1.763, 2.825)	0.650	*I*^2^ = 81.5%	Random effects model
eGFR						
Spironolactone	4	−6.545	(−9.157, −3.933)	0.000	*I*^2^ = 0.0%	Fixed effects model
Eplerenone	N/A	N/A	N/A	N/A	N/A	N/A
Finerenone	1	−2.390	(−6.214, 1.434)	0.221	—	Fixed effects model
Canrenone	N/A	N/A	N/A	N/A	N/A	N/A
Age < 65 years	1	−5.100	(−17.161, 6.961)	0.407	—	Fixed effects model
Age ≥ 65 years	2	−6.036	(−9.429, −2.643)	0.000	*I*^2^ = 0.0%	Fixed effects model
Unknown age	2	−4.872	(−9.943, 0.200)	0.060	*I*^2^ = 67.4%	Random effects model
LVEF < 40%	4	−4.837	(−7.476, −2.198)	0.000	*I*^2^ = 6.3%	Fixed effects model
LVEF ≥ 40%	1	−6.000	(−9.745, −2.255)	0.002	—	Fixed effects model
Creatinine						
Spironolactone	1	17.800	(3.100, 32.500)	0.018	—	Fixed effects model
Eplerenone	2	4.412	(1.884, 6.940)	0.001	*I*^2^ = 0.0%	Fixed effects model
Finerenone	N/A	N/A	N/A	N/A	N/A	N/A
Canrenone	N/A	N/A	N/A	N/A	N/A	N/A
Age < 65 years	N/A	N/A	N/A	N/A	N/A	N/A
Age ≥ 65 years	3	6.537	(−1.354, 14.428)	0.104	*I*^2^ = 39.9%	Random effects model
Unknown age	N/A	N/A	N/A	N/A	N/A	N/A
LVEF < 40%	1	4.500	(1.947, 7.053)	0.001	—	Fixed effects model
LVEF ≥ 40%	2	9.716	(−7.654, 27.087)	0.273	*I*^2^ = 55.3%	Random effects model
Creatinine elevation events						
Spironolactone	2	1.445	(1.163, 1.796)	0.001	*I*^2^ = 0.0%	Fixed effects model
Eplerenone	2	1.918	(0.801, 4.590)	0.143	*I*^2^ = 0.0%	Fixed effects model
Finerenone	1	1.671	(1.096, 2.548)	0.017	—	-
Canrenone	N/A	N/A	N/A	N/A	N/A	N/A
Age < 65 years	2	1.481	(0.715, 3.072)	0.291	*I*^2^ = 0.0%	Fixed effects model
Age ≥ 65 years	3	1.514	(1.245, 1.840)	0.000	*I*^2^ = 0.0%	Fixed effects model
Unknown age	N/A	N/A	N/A	N/A	N/A	N/A
LVEF < 45%	2	1.481	(0.715, 3.072)	0.291	*I*^2^ = 0.0%	Fixed effects model
LVEF ≥ 45%	3	1.514	(1.245, 1.840)	0.000	*I*^2^ = 0.0%	Fixed effects model
Composite renal outcome						
Spironolactone	3	1.785	(1.299, 2.453)	0.000	*I*^2^ = 0.0%	Fixed effects model
Eplerenone	3	0.887	(0.618, 1.275)	0.518	*I*^2^ = 0.0%	Fixed effects model
Finerenone	2	1.169	(0.385, 3.549)	0.783	*I*^2^ = 79.5%	Random effects model
Canrenone	N/A	N/A	N/A	N/A	N/A	N/A
Age < 65 years	1	2.000	(0.068, 58.583)	0.687	—	Fixed effects model
Age ≥ 65 years	6	1.300	(0.882, 1.914)	0.185	*I*^2^ = 57.2%	Random effects model
Unknown age	1	0.616	(0.249, 1.525)	0.294	—	Fixed effects model
LVEF < 40%	5	0.858	(0.614, 1.198)	0.369	*I*^2^ = 0.0%	Fixed effects model
LVEF ≥ 40%	3	1.839	(1.413, 2.394)	0.000	*I*^2^ = 0.0%	Fixed effects model

Abbreviations: eGFR, estimated glomerular filtration rate; LVEF, Left Ventricular Ejection Fraction; RR, risk ratio; WMD, weighted mean difference.

### 3.1 Effect of mineralocorticoid receptor antagonists (MRAs) on cardiovascular outcomes in heart failure patients

#### 3.1.1 All-cause mortality

**Figure 2:**
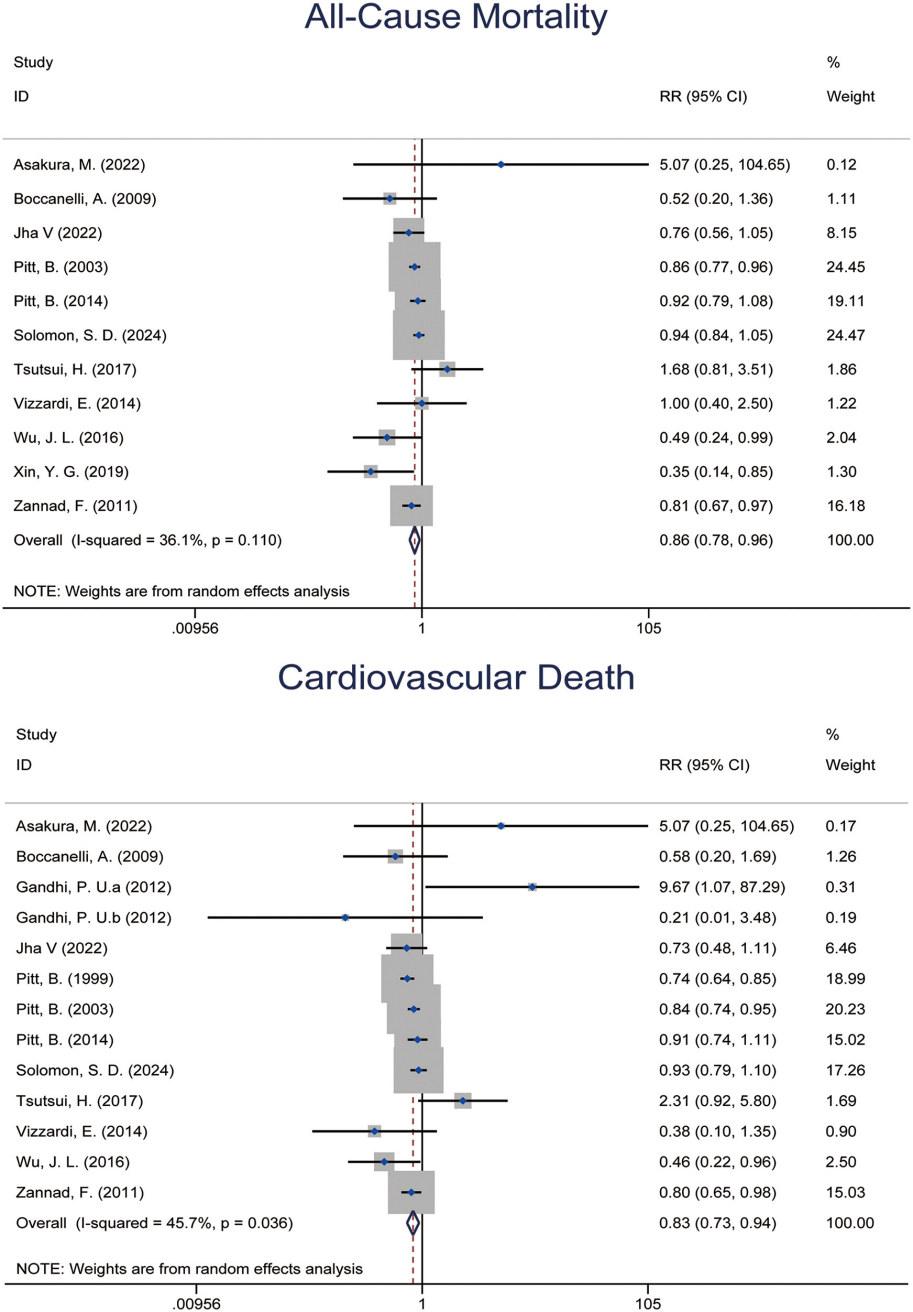
Forest plot of meta-analysis for All-cause mortality and cardiovascular mortality.

Eleven RCTs (6, 7, 10, 14, 22, 23, 31, 37, 39–41) involving 20,802 patients reported the effect of MRAs on all-cause mortality. Meta-analysis showed that MRAs significantly reduced the risk of all-cause mortality by 13.8% [RR: 0.862 (95% CI: 0.778, 0.956), *p* = 0.005; *I*^2^ = 36.1%] (Figure 2). Subgroup analysis found that MRAs significantly reduced all-cause mortality in HFrEF patients [RR: 0.861 (95% CI: 0.784, 0.947), *P* = 0.002; *I*^2^ = 21.0%], with eplerenone showing a particularly significant effect, reducing the risk of all-cause mortality by 14.9% [RR: 0.851 (95% CI: 0.743, 0.974), *P* = 0.020; *I*^2^ = 26.3%].

#### 3.1.2 Cardiovascular mortality

Twelve RCTs (5–7, 10, 14, 22, 23, 29, 31, 37, 39, 40) with 22,337 patients showed that MRAs significantly reduced the risk of cardiovascular mortality (Figure 2). The pooled effect from meta-analysis yielded an RR of 0.828 [95% CI: 0.732, 0.937], *p* = 0.003; *I*^2^ = 45.7%. This indicated a 17.2% reduction in cardiovascular mortality compared to placebo or standard treatment. Subgroup analysis revealed that the benefit of MRAs was more pronounced in older patients (mean age ≥65 years) [RR: 0.841 (95% CI: 0.716, 0.987), *P* = 0.034; *I*^2^ = 53.4%].

#### 3.1.3 Heart failure hospitalization

**Figure 3:**
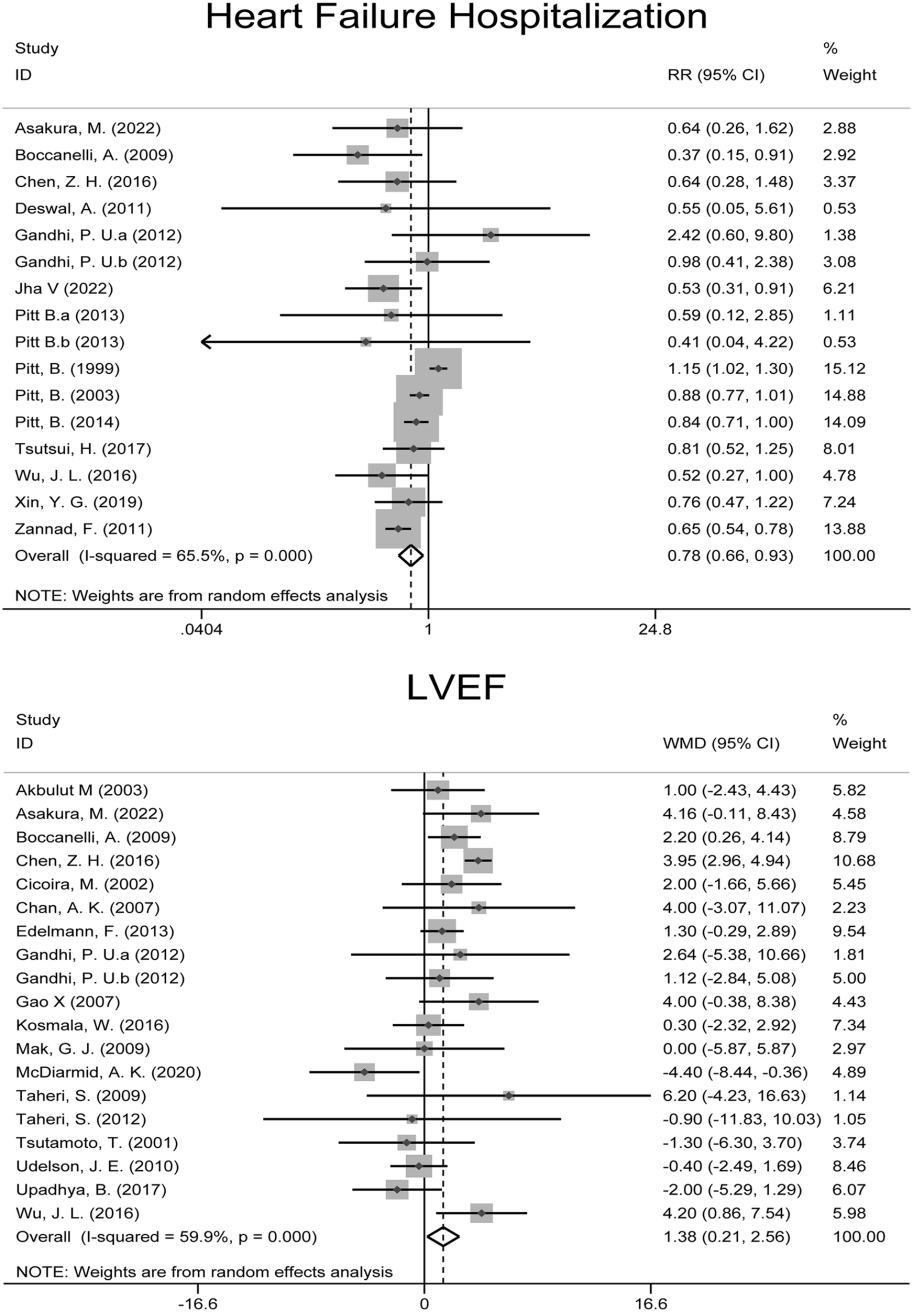
Forest plot of meta-analysis for heart failure hospitalization and LVEF outcomes.

Fourteen RCTs (5–7, 10, 22, 23, 25, 27, 29, 31, 35, 37, 40, 41) with 16,434 patients reported the incidence of heart failure hospitalization. Meta-analysis showed that MRAs significantly reduced the risk of hospitalization for heart failure [RR: 0.780 (95% CI: 0.657, 0.926), *p* = 0.005; *I*^2^ = 65.5%] (Figure 3). Notably, MRAs reduced the hospitalization rate by 22%. Further subgroup analysis revealed that MRAs had a more pronounced benefit in patients with baseline LVEF ≥ 40% [RR: 0.823 (95% CI: 0.702, 0.966), *P* = 0.017; *I*^2^ = 0.0%]. Additionally, eplerenone demonstrated superior efficacy in reducing heart failure hospitalization risk [RR: 0.738 (95% CI: 0.611, 0.893), *P* = 0.002; *I*^2^ = 44.3%].

#### 3.1.4 LVEF

Eighteen RCTs (21–26, 28–30, 32–34, 36, 38, 40, 42–44) with 2,182 patients reported LVEF outcomes. Meta-analysis showed that MRAs significantly improved LVEF in heart failure patients [WMD: 1.384 (95% CI: 0.208, 2.559), *p* = 0.021; *I*^2^ = 59.9%] (Figure 3).

### 3.2 Effect of mineralocorticoid receptor antagonists on renal-related outcomes in patients with heart failure

#### 3.2.1 Hyperkalemia

**Figure 4:**
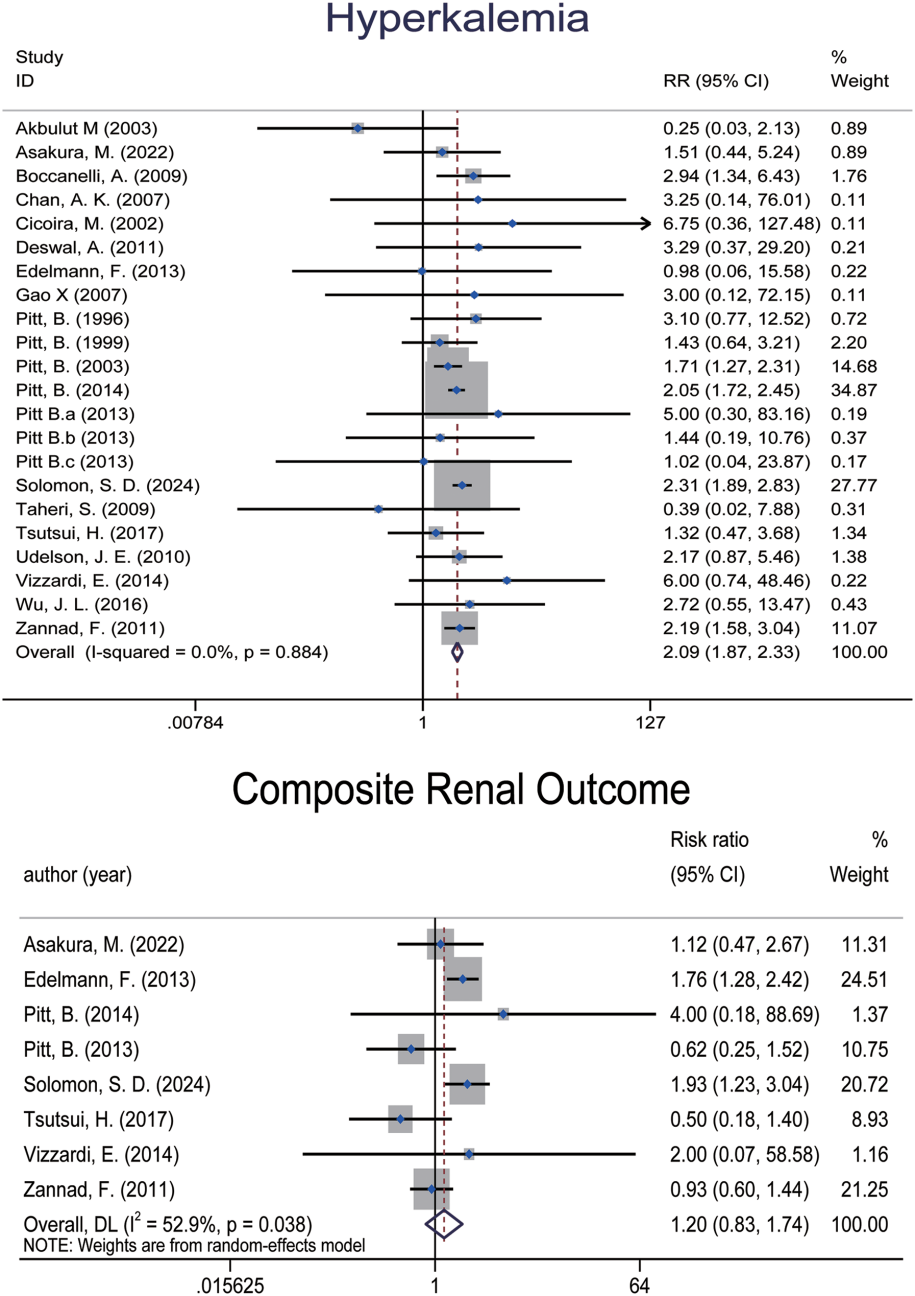
Forest plot of meta-analysis for hyperkalemia events and composite renal outcome.

Twenty RCTs (5–7, 10, 14, 20–24, 26–28, 30, 35, 37–40, 42) involving 23,387 patients reported the incidence of hyperkalemia. Meta-analysis found a significant increase in the risk of hyperkalemia associated with MRAs [RR: 2.086 (95% CI: 1.872, 2.325), *p* = 0.000; *I*^2^ = 0.0%] (Figure 4).

#### 3.2.2 Composite renal outcome

Eight RCTs (6, 7, 14, 22, 28, 35, 37, 39), encompassing 13,559 patients, reported composite renal outcomes (Figure 4). The pooled analysis demonstrated an elevated risk of composite renal adverse events in patients treated with MRAs [RR: 1.202 (95% CI: 0.830, 1.743), *p* = 0.330; *I*^2^ = 52.9%], though the result did not reach statistical significance. Subgroup analyses suggested that the increased risk was significant only in the spironolactone group and in patients with LVEF > 40%, while no significant differences were observed in other subgroups.

#### 3.2.3 Changes in eGFR

**Figure 5:**
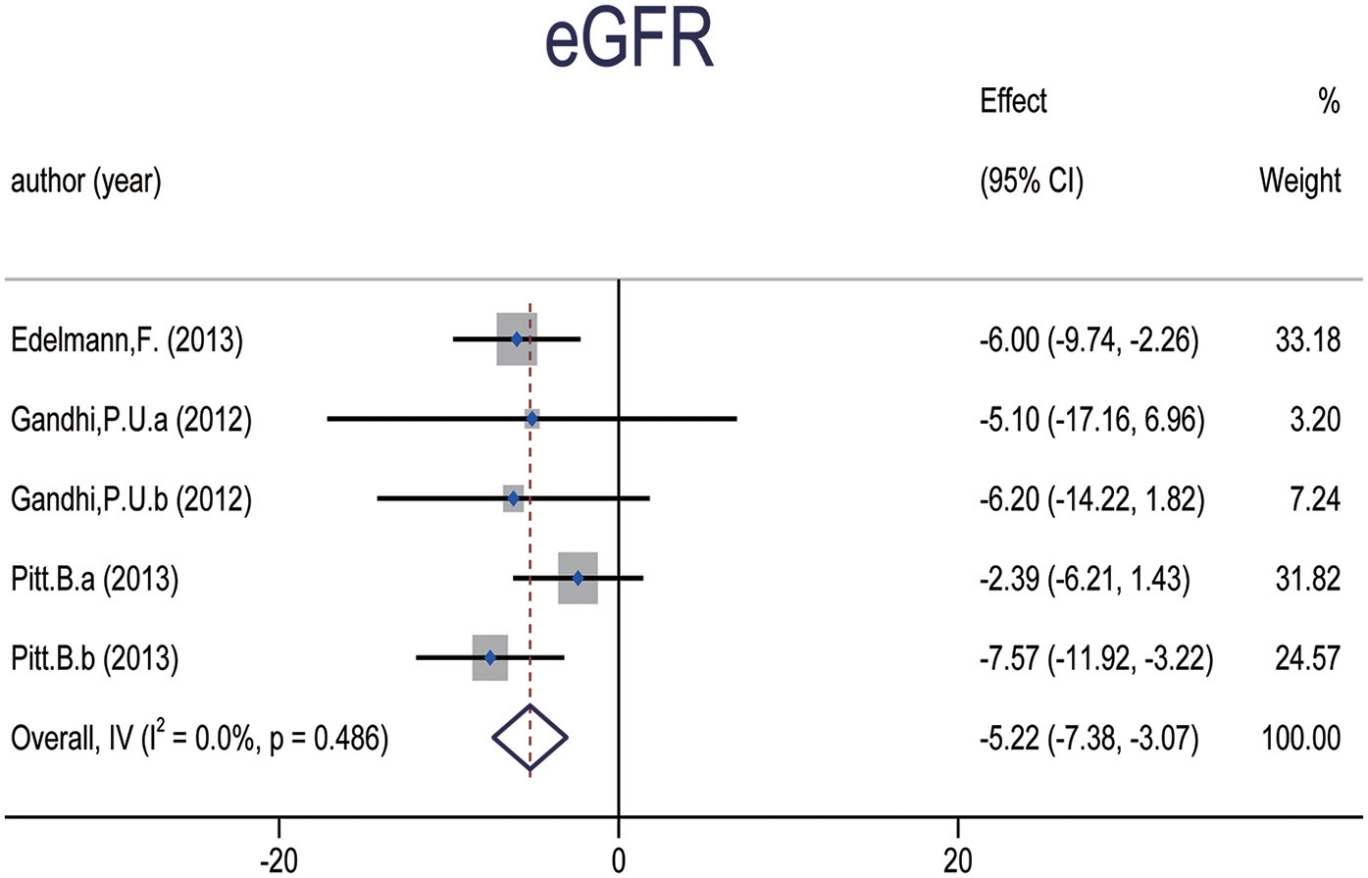
Forest plot of meta-analysis for eGFR.

Three studies (28, 29, 35), comprising five data sets and involving a total of 910 patients, reported changes in estimated glomerular filtration rate (eGFR) following MRA treatment. Meta-analysis showed a significant reduction in eGFR associated with MRA therapy [WMD: −5.223 (95% CI: −7.380, −3.066), *p* = 0.000; *I*^2^ = 0.0%] (Figure 5). Subgroup analyses revealed that spironolactone was primarily responsible for this effect, whereas finerenone did not show a significant impact on eGFR. No meaningful differences were observed across LVEF subgroups.

#### 3.2.4 Changes in Serum creatinine and risk of creatinine elevation

**Figure 6:**
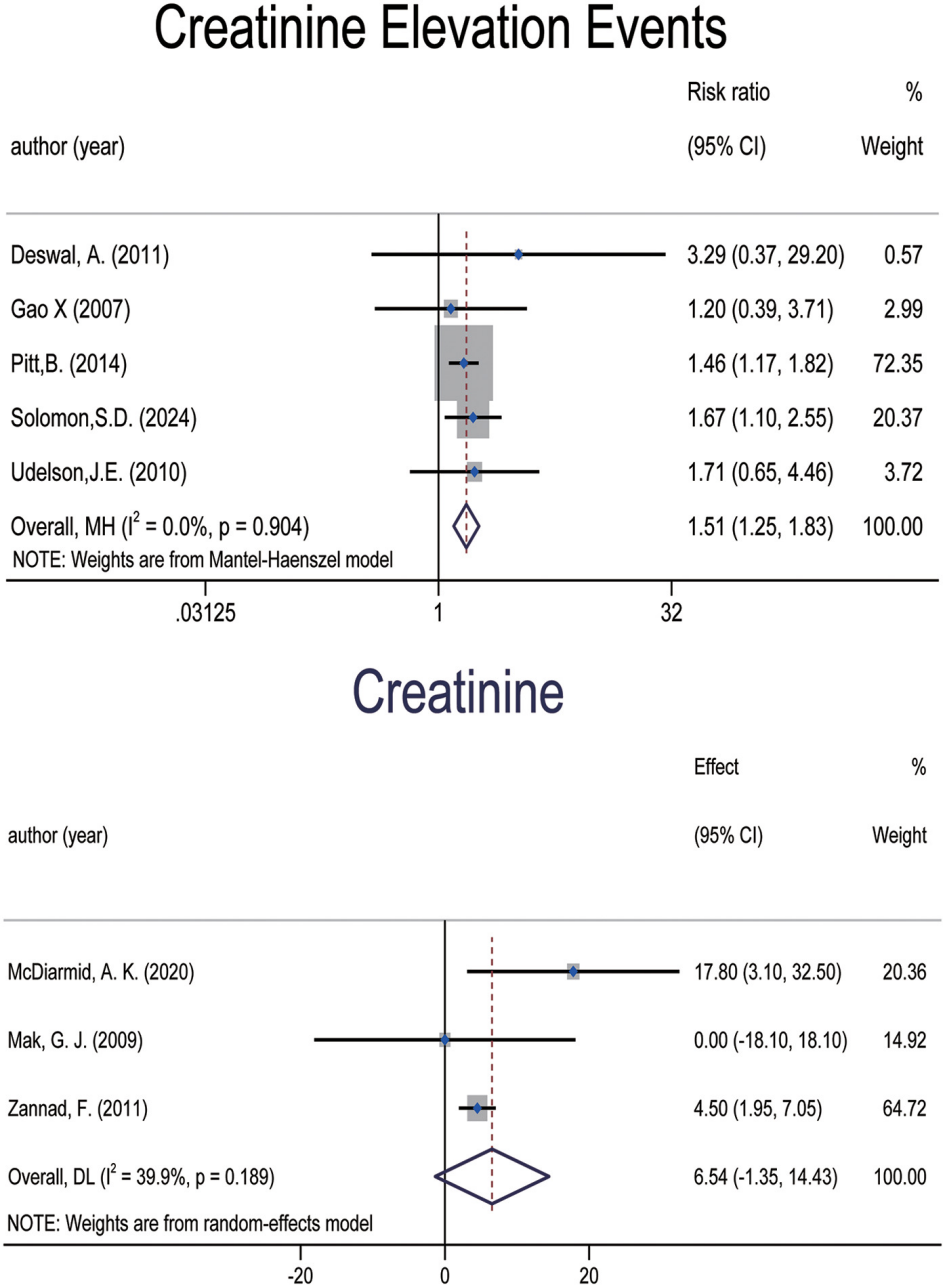
Forest plot of meta-analysis for changes in serum creatinine and risk of creatinine elevation.

Three studies (6, 34, 36), involving a total of 2,821 patients, reported serum creatinine levels as a continuous outcome (Figure 6). The pooled result indicated a trend toward elevated creatinine levels in patients receiving MRAs [WMD: 6.537; (95% CI: −1.354, 14.428), *p* = 0.104; *I*^2^ = 39.9%], although this did not reach statistical significance. Both spironolactone and eplerenone contributed to the observed increase, with a more pronounced effect in patients with LVEF < 40%. Due to the lack of data on finerenone and canrenone in these studies, we could not assess potential differences among MRA subtypes in terms of their impact on serum creatinine.

However, when considering creatinine elevation as a dichotomous outcome, five studies (7, 14, 27, 30, 38), involving 9,616 patients, were included in the analysis (Figure 6). The results indicated a significant increase in the risk of creatinine elevation with MRA use [RR: 1.512; (95% CI: 1.252, 1.825), *p* = 0.000; *I*^2^ = 0.0%]. Given the limited number of included studies and the presence of a large trial that used LVEF ≥ 45% as an inclusion criterion, we revised the original LVEF cutoff in this subgroup analysis to 45%. The results showed that the risk of elevated creatinine was more pronounced in the spironolactone and finerenone subgroups, as well as in older patients and those with LVEF ≥ 45%. In contrast, the other subgroup findings were less conclusive.

### 3.3 Publication bias

**Figure 7:**
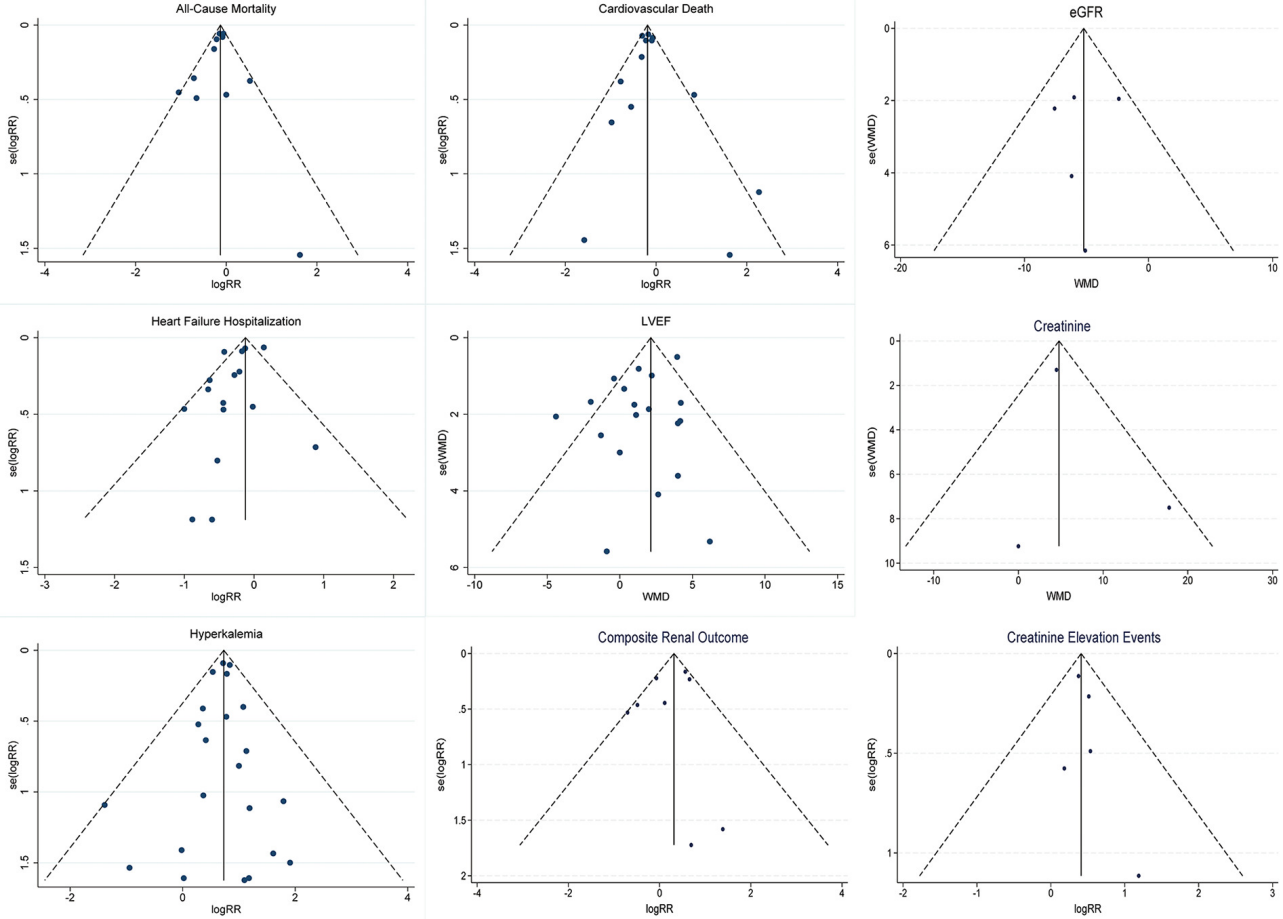
Funnel plots for outcome metrics.

The funnel plot (Figure 7) and Egger's asymmetry test (Supplementary Table S3) indicate no significant publication bias among the included trials in this study.

### 3.4 GRADE evaluation of evidence for MRA effects

**Figure 8:**
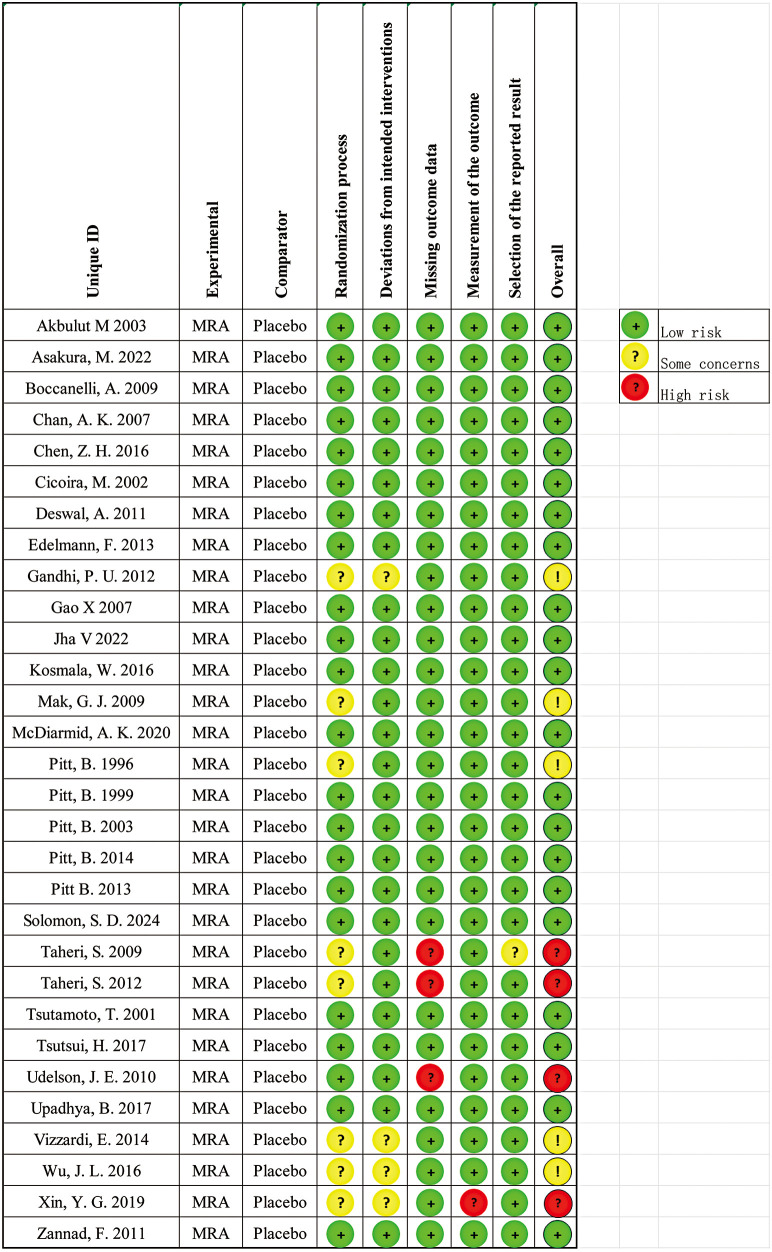
ROB2 assessment of each included trial.

The quality of evidence is summarized in Table 3. Based on the risk-of-bias evaluation using the ROB2 tool (Figure 8), the overall risk in the included RCTs ranged from low to some concerns. Most studies rated as having some concerns were due to inadequate or unreported allocation concealment during the randomization process. A few studies, such as Taheri et al. (42) and Taheri et al. (43), were judged at high risk of bias primarily because of small sample sizes and a high proportion of missing data, despite the absolute number of dropouts being low. Given their limited sample size and minimal weight in the pooled effect estimates, these high-risk studies are unlikely to materially affect the overall evidence quality.

**Table 3:** GRADE assessment of evidence for MRA effects on heart failure.

Certainty assessment							№ of patients		Effect		Certainty	Importance
№ of studies	Study design	Risk of bias	Inconsistency	Indirectness	Imprecision	Publication bias	[intervention]	[comparison]	Relative (95% CI)	Absolute (95% CI)		
All-cause mortality												
11	Randomised trials	No serious risk of bias	No serious inconsistency	No serious indirectness	No serious indirectness	Undetected	10,424	10,268	RR (0.78,0.96)	—	⨁⨁⨁⨁ HIGH	CRITICAL
Cardiovascular death												
12	Randomised trials	No serious risk of bias	No serious inconsistency	no serious Indirectness	No serious indirectness	Undetected	11,115	11,222	RR (0.73,0.94)	—	⨁⨁⨁⨁ HIGH	CRITICAL
Heart failure hospitalization												
14	Randomised trials	Serious	Serious	No serious indirectness	No serious imprecision	Undetected	8,224	8,210	RR (0.66,0.93)	—	⨁⨁○○ LOW	CRITICAL
Hyperkalemia												
20	Randomised trials	Serious	No serious inconsistency	No serious indirectness	No serious imprecision	Undetected	11,896	11,491	RR (1.87,2.33)	—	⨁⨁⨁○ MODERATE	IMPORTANT
LVEF												
18	Randomised trials	Serious	Serious	No serious indirectness	No serious imprecision	Undetected	1,085	1,097	WMD (0.21,2.56)	—	⨁⨁○○ LOW	IMPORTANT
Composite renal outcome												
8	Randomised trials	No serious risk of bias	Serious	No serious indirectness	Serious	Undetected	6,876	6,683	RR (0.83,1.74)	—	⨁⨁○○ LOW	IMPORTANT
eGFR												
3	Randomised trials	Serious	No serious inconsistency	No serious indirectness	No serious imprecision	Undetected	531	379	WMD (−7.38, −3.07)	—	⨁⨁⨁○ MODERATE	IMPORTANT
Creatinine												
3	Randomised trials	Serious	No serious inconsistency	No serious indirectness	Serious	Undetected	1,407	1,414	WMD (−1.35,14.43)	—	⨁⨁○○ LOW	IMPORTANT
Creatinine elevation events												
5	Randomised trials	Serious	No serious inconsistency	No serious indirectness	No serious imprecision	Undetected	4,815	4,801	RR (1.25,1.83)	—	⨁⨁⨁○ MODERATE	IMPORTANT

CI, confidence interval; WMD, weighted mean difference.

We evaluated the certainty of evidence for key outcomes using the GRADE approach. For all-cause mortality, the evidence was rated as high quality due to low risk of bias, moderate heterogeneity (*I*^2^ = 36.1%), and a precise effect estimate (RR = 0.862; 95% CI: 0.778–0.956). Similarly, cardiovascular mortality showed high-certainty evidence with a significant reduction in risk (RR = 0.828; 95% CI: 0.732–0.937) and low risk of bias. For heart failure hospitalization, the evidence was downgraded due to moderate heterogeneity (*I*^2^ = 65.5%) and the presence of high-risk studies; however, the effect estimate remained statistically significant (RR = 0.780; 95% CI: 0.657–0.926). The use of MRAs was associated with a significantly increased risk of hyperkalemia (RR = 2.086; 95% CI: 1.872–2.325), and the certainty of this evidence was rated high owing to the absence of heterogeneity (*I*^2^ = 0%).

Regarding LVEF, the evidence was rated as moderate quality due to moderate heterogeneity (*I*^2^ = 59.9%) and some risk of bias. Nonetheless, the effect remained statistically significant (WMD = 1.384; 95% CI: 0.208–2.559). In terms of renal outcomes, the evidence for composite renal events was rated as low quality due to inconsistency (nearly half of the included studies showed effect estimates crossing the null line) and imprecision in effect direction. Other renal outcomes, such as eGFR reduction and creatinine elevation, were supported by moderate-certainty evidence, downgraded for limitations in randomization reporting or missing data. Evidence for creatinine change was further downgraded due to imprecision and risk of bias in one study with a null effect.

Overall, the evidence supports the beneficial effects of MRAs on major cardiovascular outcomes in heart failure, with high confidence in most findings. However, limitations remain for outcomes such as HF hospitalization, LVEF improvement, and renal endpoints including creatinine elevation and composite renal events.

### 3.5 Sensitivity analysis

**Figure 9:**
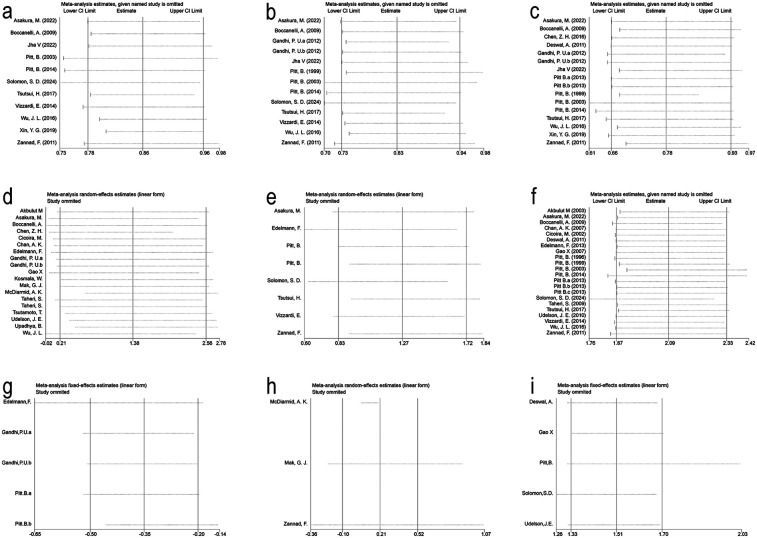
Sensitivity analysis for Key outcomes. **(a)** All-Cause Mortality. **(b)** Cardiovascular Death. **(c)** Heart Failure Hospitalization. **(d)** Left Ventricular Ejection Fraction. **(e)** Composite Renal Outcome. **(f)** Hyperkalemia. **(g)** eGFR. **(h)** Creatinine. **(i)** Creatinine Elevation Events.

To assess the robustness of our findings, we conducted sensitivity analyses. We first explored heterogeneity across studies and then performed leave-one-out analyses to determine the influence of individual studies on pooled results. Higher heterogeneity was observed in analyses of heart failure hospitalization, LVEF, and composite renal outcomes (*I*^2^ = 65.5%, 59.9%, and 52.9%, respectively), suggesting potential influence from certain studies. To further investigate, we examined funnel plots and conducted Egger's tests to detect possible publication bias or other sources of systematic bias. The funnel plots showed no notable asymmetry, and Egger's test did not reveal significant bias, indicating that heterogeneity was unlikely to be driven by publication bias. Moreover, no single study was found to significantly alter the overall estimates upon exclusion (Figure 9), confirming the stability of the meta-analytic results.

## 4 Discussion

Compared with placebo or usual care, MRA therapy in patients with heart failure resulted in a 13.8% reduction in all-cause mortality, a 17.2% reduction in cardiovascular mortality, and a 22% reduction in HF hospitalization. Additionally, treatment improved LVEF, indicating enhanced cardiac systolic function. However, it also led to increased risks of hyperkalemia, decreased eGFR, and elevated serum creatinine.

The pathophysiology of heart failure involves multiple mechanisms, including myocardial dysfunction, neurohormonal activation, hemodynamic abnormalities, and extracellular matrix remodeling (45). The core issue in heart failure is the reduced cardiac output, leading to hemodynamic disturbances and triggering compensatory mechanisms such as the Frank-Starling mechanism, activation of the sympathetic nervous system, and the renin-angiotensin-aldosterone system (RAAS) (46). While these compensatory mechanisms help maintain circulatory stability in the short term, long-term activation can lead to myocardial apoptosis and ventricular remodeling, ultimately worsening cardiac function (47).

Early animal model studies have confirmed that aldosterone, as an MR agonist, promotes vascular inflammatory cell infiltration, myocardial interstitial fibrosis, and ventricular remodeling (48–52), which further leads to myocardial, aortic fibrosis, and renal sclerosis. Spironolactone, one of the earliest MRAs, is a non-selective MR antagonist that can bind to multiple steroid receptors and inhibit fibrosis by blocking aldosterone-mediated collagen synthesis (53–57). Eplerenone, a highly selective mineralocorticoid receptor antagonist, has also demonstrated significant therapeutic effects in subsequent clinical trials.

Several large-scale randomized controlled trials have established the clinical foundation for MRA therapy in heart failure. In the RALES trial (5), it was conclusively demonstrated that spironolactone significantly reduced the risk of progressive heart failure-related mortality and sudden cardiac death. In the EPHESUS trial, which involved 3,319 heart failure patients, spironolactone significantly reduced all-cause mortality, cardiovascular mortality, and the risk of cardiovascular-related hospitalizations in patients with LVEF ≤ 40% (58), a result corroborated by our meta-analysis. The EMPHASIS-HF trial, led by Zannad F. in 2011, further demonstrated that eplerenone significantly reduced hospitalization risk in heart failure patients, offering a new therapeutic option for heart failure and expanding its use in patients with mild heart failure. Furthermore, a prospective cohort study showed that eplerenone significantly reduced the risk of cardiovascular death and all-cause mortality (59), a finding also confirmed in this study. However, the preliminary results of the TOPCAT trial—a large randomized controlled trial—indicated that spironolactone did not significantly reduce the overall incidence of the composite outcome of cardiovascular mortality, cardiac arrest, or heart failure hospitalization. However, *post-hoc* analysis revealed that spironolactone still demonstrated significant clinical benefit in patients from the Americas (7).

Finerenone, a novel non-steroidal selective mineralocorticoid receptor antagonist, has garnered wider attention due to its benefits in chronic kidney disease and diabetes patients and its ability to reduce cardiovascular event risk in large studies (6, 10). An animal study on the molecular basis of finerenone's antifibrotic activity, via selective MR cofactor modulation, showed that, compared to steroidal MRAs, the fibrotic and cardiac macrophage infiltration induced by isoproterenol was significantly blocked only in mice treated with finerenone. This study also indicated that finerenone treatment significantly improved the overexpression of cardiac TNX (a key regulator of collagen expression) induced by isoproterenol. The selective inhibition of TNX by finerenone may explain its unique antifibrotic properties. This aligns with the ARTS-HF trial (60, 61) published in 2016, which suggested that finerenone's selective MR modulation could block harmful gene activation even in the absence of aldosterone and may offer advantages over steroidal MRAs (62). MR knockout (MRKO) studies in myocardial cells (63) demonstrated the benefits of MR antagonism in improving cardiac remodeling and reducing systolic dysfunction. Furthermore, preclinical studies indicate that, at equivalent natriuretic doses, finerenone shows a more pronounced effect on reducing ventricular hypertrophy than eplerenone (64).

Several large-scale randomized controlled trialsand related meta-analyses have demonstrated the benefits of MRAs in preventing adverse cardiovascular events in heart failure patients, with the FINEARTS-HF trial providing more definitive evidence of finerenone's efficacy in heart failure patients (46–48).

While previous meta-analyses on MRAs in heart failure mainly focused on large RCTs (>1,000 patients), they often lack comprehensive subgroup exploration. Our study includes almost all relevant RCTs from multiple databases, including the recent FINEARTS-HF trial, enhancing result comprehensiveness and representation. Additionally, we assessed overall efficacy across different MRAs and further explored variation in effect by patient subgroups. Notably, Lavalle et al. (65), through network meta-analysis, highlighted variable efficacy of therapies like ARNI, SGLT2i, and vericiguat across high-risk subgroups (CKD, diabetes, women, NYHA III/IV), emphasizing the concept of stratified efficacy. This finding resonates with our observed MRA effect heterogeneity and reinforces the importance of advancing personalized management strategies in heart failure therapy.

However, we also noted that 13.6% of the patients in the FINEARTS-HF trial were already receiving SGLT2 inhibitors at baseline. Although subgroup analysis indicated that finerenone's efficacy was significant regardless of whether SGLT2 inhibitors were used, more data is needed to evaluate the broader applicability of this conclusion. Similar to the *post-hoc* analysis of the TOPCAT trial, while finerenone did not significantly improve outcomes in HFpEF patients in the FINEARTS-HF trial, this result does not directly negate its potential benefits in this population. As shown in our meta-analysis, although no significant cardiovascular outcome benefits were observed in the finerenone subgroup analysis, the limited number of studies included in this subgroup means we cannot easily conclude its ineffectiveness. Therefore, caution should be exercised when interpreting the finerenone subgroup results in this meta-analysis.

While MRAs significantly improve outcomes and provide clinical benefits in heart failure patients, it is important to note that diuretics, commonly used in heart failure treatment, can increase the risk of hypokalemia. Although MRAs reduce the occurrence of hypokalemia, they are associated with an increased risk of hyperkalemia. Both hyperkalemia and hypokalemia can lead to serious complications and pose a risk to patient safety (66).

In this meta-analysis, we paid particular attention to renal-related adverse events and classical markers of renal function following MRA use. Relevant data were extracted and analyzed from the included studies. Our results revealed that MRA therapy in heart failure patients was associated with increased risks of eGFR decline, serum creatinine elevation, worsening renal function, and renal injury. In this study, the number of studies reporting renal outcomes was relatively limited, and sample sizes were small, thus the meta-analysis had low statistical power, resulting in wide confidence intervals and greater uncertainty. While large-scale trials such as *FIDELIO-DKD* (12) and *FIGARO-DKD* (13) demonstrated the renoprotective effects of finerenone in patients with type 2 diabetes—marked by reductions in albuminuria and even potential reversal of renal impairment—our findings suggest that MRAs, when used in heart failure populations, may exacerbate renal dysfunction. The observed elevations in creatinine and reductions in eGFR could reflect either functional changes, due to aldosterone blockade and resultant afferent arteriolar constriction, or actual structural damage. Notably, our results regarding eGFR reduction are consistent with those reported by Jhund et al. (67), underscoring the need for vigilant renal monitoring during MRA therapy in heart failure patients. Nonetheless, we believe the findings offer clinical insight. This finding also highlights the need for careful renal monitoring in heart failure patients treated with MRAs, and calls for more large-scale prospective RCTs focused on renal endpoints to further validate efficacy and safety.

It is noteworthy that in the subgroup analysis of patients with reduced ejection fraction (HFrEF), the heterogeneity was modest (*I*^2^ = 21%), approaching the predefined threshold of 20%. Consequently, we initially applied a random-effects model to account for potential variability across studies, in accordance with the Cochrane Handbook, which recommends a random-effects approach when *I*^2^ ≥ 20%. However, subsequent sensitivity analyses revealed that no single study unduly influenced the pooled estimate. Moreover, the results remained consistent across different statistical models, supporting the robustness of our findings. Given this, we believe that the fixed-effects model is also justifiable—particularly in low-heterogeneity scenarios where no substantial between-study bias is detected—as it provides more precise estimates of treatment effects. The fixed-effects analysis yielded an RR of 0.861 [95% CI: 0.780–0.947], *p* = 0.002, reaffirming the stability and consistency of our conclusions.

In conclusion, although the *I*^2^ value in the subgroup analysis was close to 20%, after careful consideration of different model choices, we believe that the fixed-effects model was reasonable. We cautiously selected this model, ensuring the statistical reliability and clinical significance of our final conclusion based on the characteristics of the included studies, the observed heterogeneity, and the results of sensitivity analyses. We believe this approach not only aligns with the latest Cochrane standards but is also crucial for ensuring the scientific integrity and clinical applicability of the conclusions.

### 4.1 Limitations

Despite the strengths of this study, there are several limitations: (1) Some individual trials exhibited bias risks related to randomization allocation and data missingness, which may have affected the quality of evidence for certain outcomes. Although we attempted to contact the authors of the relevant studies for additional information, we were unable to overcome this limitation; (2) In our analysis, baseline characteristics and interventions varied among trials. Some did not report NYHA classification, and despite supplementary searches, we could not obtain complete data for NYHA-based subgroup analysis. However, existing evidence supports differential efficacy of MRAs across NYHA classes, especially showing greater benefit among NYHA III–IV HFrEF patients (67). MRAs reduce sodium retention, myocardial fibrosis, and remodeling—pathological processes more pronounced in advanced disease. RALES (5), EPHESUS (10), and EMPHASIS-HF (6) all demonstrated significant reductions in mortality and hospitalization in NYHA II–IV HFrEF patients, with ∼30% mortality reduction in NYHA III–IV, and about 24% in NYHA II. Among HFpEF (primarily NYHA II–III) patients, although hospitalization is reduced, mortality reduction has not been confirmed, and current guidelines issue only a IIb recommendation (3). The 2021 ESC (68) and 2022 AHA/ACC/HFSA (3) guidelines also tailor MRA recommendations by NYHA class. The inability to perform NYHA-based analysis is thus a key limitation, and we suggest that future research should specifically explore the interaction between NYHA class and MRA efficacy.; (3) Research on finerenone and canrenone remains limited, and more high-quality trials are needed to confirm their efficacy.

## 5 Conclusions

This meta-analysis demonstrates that MRAs significantly reduce all-cause mortality, cardiovascular mortality, and the risk of hospitalization of heart failure in patients with heart failure, while also improving left ventricular ejection fraction (LVEF). However, the risk of hyperkalemia and renal-related adverse events must not be overlooked. Our meta-analysis results indicate that MRAs significantly reduce all-cause mortality, cardiovascular mortality, and hospitalization for heart failure, and also improve LVEF. However, their use requires vigilance regarding hyperkalemia and potential renal adverse events, thus dynamic monitoring of electrolytes and renal function is warranted during MRA therapy. Additionally, current studies on finerenone are scarce and small in sample size, so we cannot draw definitive conclusions on its efficacy in HFmrEF and HFpEF populations. Future large-scale RCTs are urgently needed to establish its role in individualized treatment of heart failure.
