# Supplementary material for: To MRAs treatment or not? evidence from a meta-analysis of randomized controlled trials of different MRAs on cardiovascular health in heart failure
Source: Front Cardiovasc Med. 2025 Jul 23;12:1564860. doi: 10.3389/fcvm.2025.1564860 (PMC12325361; doi:10.3389/fcvm.2025.1564860)
Supplement: Supplementary file 1 [file Table1.docx]

**Supplementary Table 1.** The detailed definitions for composite renal outcome in the article.

| **Author** | **Year** | **Outcomes** | **The detailed definitions in the article** |
| --- | --- | --- | --- |
| **Asakura, M.** | **2022** | **Renal impairment** | **None** |
| **Edelmann, F.** | **2013** | **Significant renal impairment** | **Worsening renal function is defined as worsening as reported by the physician, decrease of eGFR to below 30 mL/min/1.73 m^2^, or decrease of eGFR by more than 15 mL/min/1.73 m^2^ vs baseline.** |
| **Pitt, B.** | **2014** | **Renal death** | **None** |
|  |  |  |  |
| **Pitt, B.** | **2013** | **Worsening of renal function** | **Any increase in serum creatinine by ≥0.3 mg/dL from baseline and/or decrease in estimated glomerular filtration rate by ≥25% from baseline; includes renal failure chronic, renal injury, and renal impairment.** |
| **Solomon, S. D.** | **2024** | **Kidney composite outcome** | **The kidney composite outcome was defined as a composite of a sustained decrease in the estimated glomerular filtration rate (eGFR) of 50% or greater, a sustained decline in the eGFR to less than 15 ml per minute per 1.73 m^2^ of body surface area, or the initiation of long-term dialysis or kidney transplantation, assessed in a time-to-event analysis** |
| **Tsutsui, H.** | **2017** | **Hospitalization for worsening renal function** | **None** |
| **Vizzardi, E.** | **2014** | **Worsening renal function** | **None** |
| **Zannad, F.** | **2011** | **Renal failure** | **None** |
