# Supplementary material for: To MRAs treatment or not? evidence from a meta-analysis of randomized controlled trials of different MRAs on cardiovascular health in heart failure
Source: Front Cardiovasc Med. 2025 Jul 23;12:1564860. doi: 10.3389/fcvm.2025.1564860 (PMC12325361; doi:10.3389/fcvm.2025.1564860)
Supplement: Supplementary file 2 [file Table2.docx]

**Supplementary Table 2**. Results of Egger's and Begg's Tests for Outcome Metrics.

| Outcome Metric | Egger’s test | Begg’s test |
| --- | --- | --- |
| All-Cause Mortality | *P*=0.478 | *P*=0.484 |
| Cardiovascular Death | *P*=0.745 | *P*=0.903 |
| Heart Failure Hospitalization | *P*=0.109 | *P*=0.928 |
| Hyperkalemia | *P*=0.608 | *P*=0.933 |
| LVEF | *P*=0.114 | *P*=0.649 |
| eGFR | *P*=0.806 | *P=0.624* |
| Creatinine | *P*=0.692 | *P*=0.602 |
| Creatinine Elevation Events | *P*=0.369 | *P*=*0.327* |
| Composite Renal Outcome | *P*=*0.432* | *P*=*0.621* |

Abbreviations: eGFR: estimated glomerular filtration rate; LVEF: Left Ventricular Ejection Fraction.
